# Age-related variations in mental health and wellbeing during the first wave of the COVID-19 pandemic: a multi-country survey analysis

**DOI:** 10.3389/fpubh.2025.1550719

**Published:** 2025-05-21

**Authors:** Moréniké Oluwátóyìn Foláyan, Roberto Ariel Abeldaño Zuñiga, Nourhan M. Aly, Passent Ellakany, Oliver Chukwujekwu Ezechi, Balgis Gaffar, Olanrewaju Ibikunle Ibigbami, Ifeoma Eugenia Idigbe, Anthonia Omotola Ishabiyi, Yousef Khader, Folake Barakat Lawal, Joanne Lusher, Kessketlen Alves Miranda, Nicaise Ndembi, Bamidele Olubukola Popoola, Mir Faeq Ali Quadri, Jorma I. Virtanen, Maha El Tantawi, Annie L. Nguyen

**Affiliations:** ^1^MEHEWE Study Group, Obafemi Awolowo University, Ile-Ife, Nigeria; ^2^Department of Child Dental Health, Obafemi Awolowo University, Ile-Ife, Nigeria; ^3^Department of Clinical Sciences, Nigerian Institute of Medical Research, Lagos, Nigeria; ^4^Postgraduate Department, University of Sierra Sur, Oaxaca, Mexico; ^5^Centre for Social Data Science, Faculty of Social Sciences, University of Helsinki, Helsinki, Finland; ^6^Department of Pediatric Dentistry and Dental Public Health, Faculty of Dentistry, Alexandria University, Alexandria, Egypt; ^7^Department of Substitutive Dental Sciences, Imam Abdulrahman Bin Faisal University, Dammam, Saudi Arabia; ^8^Department of Preventive Dental Sciences, College of Dentistry, Imam Abdulrahman bin Faisal University, Dammam, Saudi Arabia; ^9^Department of Mental Health, Obafemi Awolowo University, Ile-Ife, Nigeria; ^10^Department of Sociology, University of Cincinnati, Cincinnati, OH, United States; ^11^Department of Public Health and Epidemiology, Faculty of Medicine, Jordan University of Science and Technology, Irbid, Jordan; ^12^Department of Periodontology and Community Dentistry, University of Ibadan, Ibadan, Nigeria; ^13^Provost's Group, Regent's University London, London, United Kingdom; ^14^Research Center on Physical Activity, Health, and Leisure - CIAFEL, Faculty of Sports, University of Porto, Porto, Portugal; ^15^Institute of Human Virology, University of Maryland School of Medicine, Baltimore, MD, United States; ^16^International Vaccine Institute (IVI), Africa Regional Office, Kigali, Rwanda; ^17^Department of Child Oral Health, University of Ibadan, Ibadan, Nigeria; ^18^Department of Oral Health Sciences, School of Dentistry, University of Washington, Seattle, WA, United States; ^19^Faculty of Medicine, University of Bergen, Bergen, Norway; ^20^Herbert Wertheim School of Public Health and Human Longevity Science, University of California, San Diego, San Diego, CA, United States

**Keywords:** mental health, wellbeing, wellness, global health, post-traumatic stress disorder

## Abstract

**Background:**

The COVID-19 pandemic substantially impacted mental health. This study explored age-related differences in the mental health and wellbeing of participants during the initial wave of the COVID-19 pandemic.

**Methods:**

Secondary analyses of data from 21,106 participants collected between July and December 2020 across 152 countries was conducted. Multivariable logistic regression models were used to determine the relationship between the dependent variables and age after accounting for potential confounders. The dependent variables examined were emotional distress, social isolation, sleep pattern changes, sexual activity changes, financial security, housing and food insecurity, changes in family relationships, memory complaints, and post-traumatic stress syndrome (PTSS). Age groups were considered: young adults (18–24), adults (25–49), and older individuals (≥50). The confounding variables were sociodemographic variables namely: sex (male or female), marital status (single or has had a relationship), employment status (employed, unemployed, student, or retiree), educational status (none, primary, secondary, or college/university), country of residence income (low-income countries, lower-middle-income countries, upper-middle-income countries, and high-income countries) and pandemic stringency index indicating the comprehensiveness of pandemic policies adopted at country level.

**Results:**

Of the 21,106 participants, 12,807 (60.7%) were aged 25–49. Compared to those over 50, 18–24-year-olds had significantly higher odds of increased sexual activity (AOR: 4.41), housing insecurity (AOR: 1.73), and PTSD (AOR: 3.22), but lower odds of social isolation (AOR: 0.66), food insecurity (AOR: 0.85), and memory complaints (AOR: 0.53). Similarly, 25–49-year-olds had higher odds of increased sexual activity (AOR: 2.65), housing insecurity (AOR: 2.28), food insecurity (AOR: 1.14), worsened family relationships (AOR: 1.15), and PTSD (AOR: 2.24), but lower odds of social isolation (AOR: 0.84), sleep disturbances (AOR: 0.82), and memory complaints (AOR: 0.81). Emotional distress and financial insecurity did not differ significantly across age groups.

**Conclusion:**

The study highlights age-specific variations in mental health challenges during the pandemic. The nuanced impact of age on mental wellbeing emphasizes the need for targeted interventions. Future research should explore the interplay between age, pandemic-related factors, and specific mental health outcomes to inform tailored support mechanisms for diverse age groups.

## Introduction

The COVID-19 pandemic profoundly disrupted global health and psychosocial wellbeing, amplifying pre-existing vulnerabilities and introducing novel stressors. It had a significant impact on the mental health and wellbeing of individuals and populations, especially women, adolescents, and individuals affected by social inequality (1, 2). People with pre-existing health conditions, such as HIV, expressed heightened concerns about contracting COVID-19 (1, 2). Individuals who displayed COVID-19 symptoms without undergoing testing, as well as those with connections to positive COVID-19 cases or who had experienced loss due to the pandemic, were more prone to feelings of fear, anxiety, or depression (3).

Before the pandemic, determinants of mental health included socioeconomic status, social support, employment stability, and access to healthcare (4). However, the pandemic reshaped the salience and interplay of these factors, creating a unique psychosocial landscape. The rates of these emotional and mental states were significantly high during the first wave of the pandemic (5). In addition, the pandemic induced a range of negative emotions, including frustration, anxiety, depression, boredom, loneliness, anger, and grief among individuals (6, 7). Experiencing emotional distress was linked to a higher probability of developing health issues like oral ulcers, ultimately reducing the quality of life for those affected (8–10). Furthermore, people reported alterations in sleep patterns, memory complaints, and changes in sexual activities, which could be linked to the emotional distress experienced during the pandemic (11–16).

Implementing preventive measures such as lockdowns and social distancing, while necessary to curb viral transmission, exposed many people to vulnerabilities that reduced their capacity to live meaningfully (17–19). These exposures included financial insecurity, housing instability, and food scarcity (20). These vulnerabilities further escalated the risk of emotional distress, post-traumatic stress disorders (21), strained family relationships, and a diminished quality of life (22–25) with negative impact on the wellbeing and mental health of the population (25).

COVID-19-related stress prompted individuals to embrace a range of coping strategies, including acquiring new skills, engaging in meditation or mindfulness practices, maintaining social connections through technological use such as video chats, use of social network sites, and undertaking indoor or outdoor exercises (26–28). These are arguably positive coping techniques, but some individuals turn to maladaptive coping mechanisms, such as increased tobacco smoking or alcohol consumption and the use of other psychoactive substances (29, 30). Social isolation heightened the likelihood of experiencing poor mental health and adopting maladaptive coping mechanisms (31–34). At the same time, having access to social support played a pivotal role in enhancing mental wellbeing by reducing the risk of post-traumatic stress symptoms (35, 36), even in young people (37–41).

Sociodemographic variables significantly moderate the impact of the COVID-19 pandemic on individuals' mental health and wellbeing (42–47), including adherence to COVID-19 preventive measures (48, 49). In addition, biomedical factors, including viral load and co-morbidity status in people living with HIV, contributed to individual wellness amid the pandemic (12, 50–52). Gender disparities have been noted in the way the pandemic affects mental health (35). However, evidence regarding age-related variations in the mental health impact of the pandemic is still emerging, as most studies have not compared its effects across different age groups (53).

The impact of COVID-19 is anticipated to vary by age. Children and adolescents grappling with disruptions to their education and social interactions due to prolonged school closures, social distancing measures, and limited access to recreational activities may have faced challenges that affected their mental health (54, 55). In addition, some had to deal with distress from the economic consequences and the feeling of uncertainty about the pandemic (56). Young and middle-aged adults encountered hurdles in sustaining their livelihoods due to job losses and business closures, resulting in emotional stress and mental health issues (57, 58). Older individuals, being the most medically vulnerable out of all age groups with the highest mortality risk from COVID-19, were disproportionately affected, particularly those in nursing homes and long-term care facilities (58, 59). Furthermore, social isolation and the fear of infection may impact mental wellbeing, including memory complaints and emotional stress, in ways that are age-distinct (33, 60, 61).

Social theories addressing age-related changes in health recognize that age-based social status can influence mental health as individuals enter and exit different social roles (62–64). People's experiences of social, economic, environmental, and historical events shape their lives and mental wellbeing (65, 66). However, the impact of age on mental health can be modified by access to education and the historical context in different geographical locations (67–69). Different factors influence mental health at different stages of life through multiple pathways, including access to resources (62), the socially structured, age-based system of role allocation (70), and psychological maturity or the decline of physiological processes (62). Employment and marital status, which create social roles that structure many aspects of life (62), significantly influence mental health (71). In addition, an individual's socio-economic status can heighten the risk of poor mental health through repeated spells of lost roles and opportunities (72), with potential multiplier effects over their lifetime (73, 74). Age provides the context for mental health and wellness risk factors to play out over the life course as people develop the capabilities to live a healthy life, live in physical safety and legal security, gain knowledge, achieve economic independence, have secure living conditions, enjoy individual, family, and social life, have self-respect, and participate in decision-making.

This study examined age-related differences in mental health and wellbeing. Specifically, analyses examined the relationships between mental health and wellbeing indicators (emotional distress, social isolation, sleep pattern changes, sexual activity changes, financial security, housing and food insecurity, changes in family relationship, memory complaints and PTSS) and age groups—young adults (18–24 years old), adults (25–49 years old), and older adults (≥50 years). The hypothesis posited that there are significant differences in mental health and wellbeing indicators—emotional distress, social isolation, sleep changes, sexual activity, financial security, housing and food insecurity, family relationship changes, memory complaints, and post-traumatic stress symptoms—across age groups, with young adults (18–24), adults (25–49), and older adults (>50) each exhibiting distinct patterns of vulnerability during the first wave of the COVID-19 pandemic.

## Methods

### Study design, settings, and population

Secondary analyses were conducted on a dataset of a cross-sectional study that collected data from 21,106 individuals 18 years and older from 152 countries between June 29, 2020, and December 31, 2020. The primary study was designed to determine the mental health and wellbeing of participants during the first wave of the COVID-19 pandemic (75).

### Data collection process

The data collection process for this study was undertaken through a large-scale international collaboration involving nearly 50 researchers from multiple regions of the world. To ensure coordination and efficiency, several key strategies were implemented from the outset, one of which was having a core team of lead researchers to streamline operations. A site was designated to manage a centralized data coordinating site by being responsible for managing the global dataset and facilitating secure data handling. The structure also supported a collaborative, organized, and ethically sound data collection process (76).

The data collection process was conducted using Survey Monkey^®^, a secure online survey platform. The survey was designed to ensure anonymity, allowing respondents to review and change their responses before final submission. To prevent duplicate entries, only one submission per electronic device was permitted. There was no time limit imposed for survey completion, enabling participants to respond at their own pace. Survey links were distributed to eligible participants via email and social media platforms. This digital approach enabled global participation, facilitating broad geographic reach and cross-cultural data collection during the height of the COVID-19 pandemic (75).

### Data collection tool

Data were collected using the COVID-19 Mental Health and Wellness Survey Questionnaire (75), a validated, multidimensional instrument designed to comprehensively assess the psychosocial and mental health impacts of the COVID-19 pandemic. Comprising 57 items, the questionnaire spans nine thematic sections, including health and memory, pandemic stress, financial and lifestyle impacts, and symptoms of post-traumatic stress disorder. It has an overall Content Validity Index of 0.83. Dimensionality was explored using Multiple Correspondence Analysis, confirming the structure of key domains. Qualitative validation confirmed the questionnaire's appropriateness, particularly among well-educated respondents. The questionnaire also showed strong internal consistency with high Cronbach's alpha values for the items, indicating excellent coherence among the items within each subscale. Test-retest reliability was also acceptable, with intraclass correlation coefficients ranging from 0.71 to 0.89, reflecting moderate to strong stability over time.

A key strength of this instrument is its cross-cultural adaptability. It was translated into English, French, Arabic, Portuguese, and Spanish, enhancing its utility in diverse socio-cultural settings. Its broad scope allows it to capture a wide array of mental health and wellbeing indicators and socioeconomic vulnerabilities. The overall psychometric performance of the instrument supports its use in capturing the complex and multidimensional impacts of the COVID-19 pandemic.

### Sample size for current analyses

The extracted dataset was appropriate and adequate for statistical modeling as it ensured a minimum of 10 participants with complete responses for each independent variable. This enabled the conduct of regression analyses with a stipulated minimum significance level of 0.05 (77).

### Sampling procedure and data collection process

The primary data were acquired using a non-probability sampling method facilitated by global dissemination of the online survey tool using Survey Monkey^®^ through posts on various social media platforms (Facebook, Twitter, and Instagram), network email lists, and WhatsApp groups. Respondents were encouraged to share the link further. Details regarding the recruitment process for study participants have been published (75). Consent was obtained for study participation before accessing the questionnaire, which took an average of 11 min to complete. Data quality was enhanced by implementing best-practice measures, including IP address restrictions, allowing each participant to complete only one questionnaire. Participants retained the flexibility to edit their answers until they chose to submit. Additional information on the survey methodology is available elsewhere (75).

### Independent variable

#### Age

The study participants were classified into three age brackets: young adults (18–24-year-olds), adults (25–49-year-olds), and older individuals (≥50-year-olds). Age was determined by age at last birthday. The age groupings in the study were based on standard epidemiological practice (78). The aimed to ensure meaningful analysis while reflecting the sample's demographic characteristics. The 18–24 age group represents emerging adults (79), a distinct developmental stage with unique psychological and social transitions that can influence mental health (80). The 25–49 age group covers early to middle adulthood, with shared experiences related to work, family, and health. Individuals aged 50 and above were grouped to capture the challenges associated with older adulthood, such as increased risk of physical and mental health issues.

### Dependent variables

#### Mental wellbeing indicators

These indicators—Social isolation, sleep and sexual activity changes, financial and housing insecurity, food insecurity, and shifts in family relationships—were categorized as mental wellbeing indicators because they reflect individuals' subjective experiences and psychological responses to the pandemic, key aspects of mental health. They have a direct link to psychosocial stressors that affect overall mental health during the pandemic.

#### Socially isolation

Participants were tasked with evaluating their sense of social isolation compared to pre-COVID-19 times, choosing from response options: “same,” “less socially isolated,” or “more socially isolated.” Subsequently, the social isolation variable was categorized into two groups: “same/less socially isolated” and “more socially isolated”.

#### Change in sleep pattern

Participants were asked about alterations in their sleep patterns during the pandemic, including sleeping more, sleeping less, experiencing other changes, or no change. Responses were dichotomized into “sleeping changes present” (including sleeping more, sleeping less, or other changes) and “sleeping changes absent” (no change in sleep pattern) for subsequent statistical analysis (12).

#### Changes in sexual activity

Participants were asked about changes in sexual activity during the COVID-19 pandemic, with response options including increase, decrease, no change, or not applicable (75). Those selecting ‘not applicable' were considered not sexually active and excluded from further analyses. Those reporting an increase were classified as “increased activity” whereas the “decrease” and “no change” responses were classified as “no increased activity: decrease or same”.

#### Financial insecurity

Participants were asked whether they encountered financial loss due to the COVID-19 pandemic, with response options “yes,” “no,” or “I don't know.” The question was adapted from the Multi-Center AIDS Cohort Study (81). “Yes” responses were categorized as financial insecurity, while “No” responses were categorized as financial security.

#### Housing security

Participants were asked whether the COVID-19 pandemic led to “Loss of your housing or becoming homeless”, with response options “yes,” “no,” or “I don't know.” The question was adapted from the Multi-Center AIDS Cohort Study (81). “Yes” responses were categorized as housing insecurity, while “No” responses were categorized as housing security.

#### Food insecurity

Respondents were assessed regarding the pandemic's impact on their access to food and meals. They answered with a “yes” or “no” to questions such as experiencing hunger but refraining from eating due to a lack of money for food, cutting the size of meals, or skipping meals due to financial constraints during the COVID-19 pandemic. Any positive response to any of these three questions was categorized as experiencing food insecurity. The questions were adapted from the US Department of Agriculture Household Food Security Survey (82).

#### Changes in family relationships

Participants were asked to characterize how the quality of their relationships with family members changed during the pandemic. Response options included “becoming a lot worse,” “a little worse,” “remaining the same,” “becoming a little better,” or “a lot better.” Responses were dichotomized into “improved/remained unchanged” (remaining the same, becoming a little better, or becoming a lot better) vs. “worsened” (becoming a lot worse and becoming a little worse).

### Mental health indicators

These indicators—emotional distress, post-traumatic stress symptoms, memory complaints—have a direct association with psychological wellbeing.

#### Emotional distress

Participants were requested to specify whether they encountered any of the listed emotions throughout the pandemic: depression, anxiety, frustration/boredom, loneliness, anger, grief/sense of loss. They were required to mark a checkbox for each emotion. Individuals who marked any of the checkboxes were categorized as having experienced emotional distress during the pandemic. If no checkbox was checked, the individual was identified as having no emotional distress during the pandemic. Previous research has utilized this evaluation method (3, 6).

#### Post-traumatic stress symptoms

PTSS was assessed using the 17-item self-report PTSD checklist for civilians (83). Each item was rated on a 5-point scale with responses ranging from 1—“not at all” to 5—“extremely.” The potential total score spanned from 17 to 85, with a designated cut-off of 28 utilized to categorize responses into “no PTSS” (17–27) and “PTSS present” (28–85) (84).

#### Memory complaint

Memory complaints were evaluated utilizing the validated Memory Complaint Questionnaire (85), which comprises six questions addressing memory functioning in daily situations. Participants gauged their current performance against their pre-pandemic status, yielding a total score ranging from 7 to 35, where higher scores indicated perceived memory loss. Scores equal to or surpassing 25 signified memory impairment. Participants were categorized into two groups regarding significant memory complaints: those without significant memory complaints (scores < 25) and those with significant memory complaints (scores ≥25) (85).

### Confounders

#### Sex

Respondents were prompted to indicate their sex at birth by checking the checkbox corresponding to male or female.

#### Marital status

Participants were asked to select the checkbox representing their current marital status from options such as single, cohabiting, widow, legally married, or divorced/separated. For statistical analysis, marital status was dichotomized into single vs. a history of conjugal relationships (co-habiting, widow, legally married, divorced/separated).

#### Employment status

Employment status options were employed, unemployed, student, or retired.

#### Educational status

Respondents were instructed to choose the checkbox corresponding to the option that best reflected their highest level of education attained, with choices including none, primary, secondary, or college/university.

#### Country of residence income

Information regarding the country's income level was derived from publicly available data in the World Bank Data Bank (86). Countries were categorized into four groups based on gross national income (GNI) per capita: low-income countries (LIC) with GNI ≤ 1,035 USD in 2019, lower-middle-income countries (LMIC) with GNI between 1,036 and 4,045 USD, upper-middle-income countries (UMIC) with GNI between 4,046 and 12,535 USD, and high-income countries (HIC) with GNI ≥12,536 USD.

#### Pandemic stringency index

The pandemic stringency index was included to gauge the impact of government policies related to closure and containment, health, and economic policies (63). This index incorporates responses in 19 policy areas, reflecting variations in the degree of response. For this study, we computed and imputed the average stringency index for the participant's country of residence during the month the respondents completed the survey. The index ranges from 0 to 100, with higher values indicating more stringent COVID-19 policies for a given country. The variable was categorized into an ordinal scale (0–19.99, 20–39.99, 40–59.99, 60–79.99, and 80–100) (63).

### Data analysis

The data underwent cleaning and was subsequently imported into SPSS version 23.0 (IBM Corp., Armonk, N.Y., USA) for analysis. A descriptive analysis was conducted for all study variables. Separate multivariable logistic regressions were conducted to determine the associations between each dependent and independent variable while considering the impact of confounding variables. We assessed multicollinearity among the variables in our model using the Variance Inflation Factor (VIF) and tolerance values. The VIF values for the predictors were all below the threshold of 10, indicating that multicollinearity is not a concern in our model.

Missing data were addressed through multiple imputation for variables with the following levels of missing responses: Socially isolated: 24.6%, Changes in family relationship: 29.7%, Memory Complaint: 16.9%, Post-traumatic stress symptoms: 27.6%, Age: 13.7%, Sex: 14.2%, Education level: 13.5%, Employment status: 13.5%, Marital status: 13.5%, and stringency index: 26.2%. After applying Little's MCAR test and identifying that missing data patterns were not at random, the logistic regression method was used for the imputation of variables. Adjusted odds ratios (AOR) and their corresponding 95% confidence intervals (CI) were calculated. The threshold for statistical significance was set at 5%.

### Ethics approval

Ethics approval for the study was obtained from the Human Research Ethics Committee at the Institute of Public Health of the Obafemi Awolowo University Ile-Ife, Nigeria (HREC No: IPHOAU/12/1557), Brazil (CAAE No. 38423820.2.0000.0010), India (D-1791-uz and D-1790-uz), Saudi Arabia (CODJU-2006F) and United Kingdom (13283/10570) for the conduct of the primary study. Study participants checked a box to indicate consent before participating in the online survey. Informed consent was obtained from all participants involved in the study.

## Results

Of the 21,106 study participants, 5,182 (24.6%) were 18–24-year-olds, 12,807 (60.7%) were 25–49-year-olds, and 3,117 (14.8%) were ≥50-year-olds. The mean (SD) for the study population was 34.8 (SD 12.9). For the 18–24 age group the mean was 21.5 (SD 1.7), for the 25–49 age group the mean age was 34.5 (SD: 6.8), and for the 50–100 age group the mean age was 58.6 (SD: 7.2). The skewness for the age variable was 0.947 (Standard error 0.018).

In addition, 13,052 (61.8%) were females, 16,592 (78.6%) had university education, 12,071 (57.2%) were employed, and 9,558 (45.3%) were single. In addition, 11,388 (54.0%) participants were from LMICs. The mean age of the study participants was 34.9 (SD: 12.9) years.

Table 1 shows that respondents 18–24 years old had significantly higher odds for increase in sexual activity (AOR: 4.411; 95% CI: 3.561–5.464; *p* < 0.001), housing insecurity (AOR: 1.733; 95% CI: 1.156–2.600; *p* = 0.008), and post-traumatic stress syndrome (AOR: 3.224; 95% CI: 2.762–3.763; *p* < 0.001) than respondents ≥50years. In addition, respondents 18-24 years old had significantly lower odds of feeling socially isolated (AOR: 0.656; 95% CI: 0.577–0.746; *p* < 0.001), food insecurity (AOR: 0.847; 95% CI: 0.721–0.995; *p* = 0.044), and memory compliant (AOR: 0.532; 95% CI: 0.446–0.635; *p* < 0.001) than respondents ≥50 years during the COVID-19 pandemic.

**Table 1:** The association between mental health, mental wellbeing, and age during the first wave of the COVID-19 pandemic in 152 countries of the world (*N* = 21,106).

**Variables**	**Total *N* (%) 21,106 (100)**	**Socially isolated**		**AOR; 95% CI; *p-*value**	**Changes in sleep pattern**		**AOR; 95% CI; *p-*value**
		**Yes** ***N*** **(%) 12,075 (57.2)**	**No** ***N*** **(%) 9,031 (42.8)**		**Yes** ***N*** **(%) 5,253 (24.9)**	**No** ***N*** **(%) 15,853 (75.1)**	
**Age**							
18–24	5,182 (24.6)	2,692 (51.9)	2,490 (48.1)	0.656; 0.577–0.746; *p* < 0.001	1,555 (30.0)	3,627 (70.0)	0.924; 0.801–1.066; *p* = 0.276
25–49	12,807 (60.7)	7,361 (57.5)	5,446 (42.5)	0.842; 0.767–0.925; *p* < 0.001	2,881 (22.5)	9,926 (77.5)	0.822; 0.741–0.913; *p* < 0.001
50	3,117 (14.8)	2,022 (64.9)	1,095 (35.1)	1	817 (26.2)	2,300 (73.8)	1
**Sex**							
Females	13,052 (61.8)	7,625 (58.4)	5,427 (41.6)	1.158; 1.093–1.227; *p* < 0.001	3,569 (27.3)	9,483 (72.7)	1.326; 1.239–1.419; *p* < 0.001
Males	8,054 (38.2)	4,450 (55.3)	3,604 (44.7)	1	1,684 (20.9)	6,370 (79.1)	1
**Education level**							
No formal Educ	400 (1.9)	134 (33.5)	266 (66.5)	0.469; 0.378–0.582; *p* < 0.001	46 (11.5)	354 (88.5)	0.445; 0.325–0.611; *p* < 0.001
Primary	479 (2.3)	206 (43.0)	273 (57.0)	0.628; 0.520–0.758; *p* < 0.001	67 (14.0)	412 (86.0)	0.537; 0.412–0.699; *p* < 0.001
Secondary	3,635 (17.2)	2,084 (57.3)	1,551 (42.7)	1.017; 0.941–1.099; *p* = 0.647	1,011 (27.8)	2,624 (72.2)	0.998; 0.916–1.088; *p* = 0.967
University	16,592 (78.6)	9,651 (58.2)	6,941 (41.8)	1	4,129 (24.9)	12,463 (75.1)	1
**Employment status**							
Retiree	846 (4.0)	573 (67.7)	273 (32.3)	1.381; 1.153–1.653; *p* < 0.001	231 (27.3)	615 (72.7)	0.882; 0.727–1.071; *p* = 0.206
Student	4,995 (23.7)	2,766 (55.4)	2,229 (44.6)	1.324; 1.191–1.473; *p* < 0.001	1,551 (31.1)	3,444 (68.9)	1.106; 0.982–1.244; *p* = 0.096
Employed	12,071 (57.2)	7,142 (59.2)	4,929 (40.8)	1.296; 1.193–1.409; *p* < 0.001	2,687 (22.3)	9,348 (77.7)	0.859; 0.781–0.946; *p* = 0.002
Unemployed	3,194 (15.1)	1,594 (49.9)	1,600 (50.1)	1	784 (24.5)	2,410 (75.5)	1
**Marital status**							
Single	9,558 (45.3)	5,264 (55.1)	4,294 (44.9)	0.995; 0.930–1.064; *p* = 0.873	2,697 (28.2)	6,861 (71.8)	1.279; 1.184–1.381; *p* < 0.001
Has had a relationship	11,548 (54.7)	6,811 (59.0)	4,737 (41.0)	1	2,556 (22.1)	8,992 (77.9)	1
**Country income**							
LIC	509 (2.4)	260 (51.1)	249 (48.9)	0.523; 0.434–0.629; *p* < 0.001	109 (21.4)	400 (78.6)	0.745; 0.596–0.932; *p* = 0.010
LMIC	11,388 (54.0)	5,797 (50.9)	5,591 (49.1)	0.653; 0.608–0.701; *p* < 0.001	2,366 (20.8)	9,022 (79.2)	0.672; 0.620–0.728; *p* < 0.001
UMIC	4,166 (19.7)	2,750 (66.0)	1,416 (34.0)	0.999; 0.915–1.092; *p* = 0.989	1,379 (33.1)	2,787 (66.9)	1.210; 1.104–1.326; *p* < 0.001
HIC	5,043 (23.9)	3,268 (64.8)	1,775 (35.2)	1	1,399 (27.7)	3,644 (72.3)	1
Stringency index	47.3 (57.8)	49.6 (27.6)	44.1 (27.8)	1.005; 1.004–1.006; *p* < 0.001	49.0 (29.2)	46.7 (27.3)	1.000; 0.999–1.001; *p* = 0.895
**Age**							
18–24	5,182 (24.6)	994 (19.2)	4,188 (80.8)	4.411; 3.561–5.464; *p* < 0.001	4,687 (90.4)	495 (9.6)	0.964; 0.770–1.206; *p* = 0.747
25–49	12,807 (60.7)	1,805 (14.1)	11,002 (85.9)	2.651; 2.217–3.169; *p* < 0.001	11,621 (90.7)	1,186 (9.3)	1.115; 0.939–1.323; *p* = 0.214
50+	3,117 (14.8)	168 (5.4)	2,949 (94.6)	1	2,901 (93.1)	216 (6.9)	1
**Sex**							
Females	13,052 (61.8)	1,564 (12.0)	11,488 (88.0)	0.622; 0.573–0.674; *p* < 0.001	11,977 (91.8)	1,075 (8.2)	0.748; 0.678–0.825; *p* < 0.001
Males	8,054 (38.2)	1,403 (17.4)	6,651 (82.6)	1	7,232 (89.8)	822 (10.2)	1
**Education level**							
No formal Educ	400 (1.9)	58 (14.5)	342 (85.5)	0.910; 0.680–1.219; *p* = 0.528	375 (93.8)	25 (6.3)	0.472; 0.311–0.715; *p* < 0.001
Primary	479 (2.3)	74 (15.4)	405 (84.6)	1.050; 0.810–1.360; *p* = 0.713	443 (92.5)	36 (7.5)	0.689; 0.486–0.976; *p* = 0.036
Secondary	3,635 (17.2)	588 (16.2)	3,047 (83.8)	1.097; 0.987–1.220; *p* = 0.087	3,240 (89.1)	395 (10.9)	1.221; 1.078–1.384; *p* = 0.002
University	16,592 (78.6)	2,247 (13.5)	14,345 (86.5)	1	15,151 (91.3)	1,441 (8.7)	1
**Employment status**							
Retiree	846 (4.0)	40 (4.7)	806 (95.3)	0.790; 0.549–1.137; *p* = 0.204	792 (93.6)	54 (6.4)	0.492; 0.355–0.682; *p* < 0.001
Student	4,995 (23.7)	859 (17.2)	4,136 (82.8)	0.988; 0.853–1.143; *p* = 0.867	4,542 (909)	453 (9.1)	0.549; 0.465–0.648; *p* < 0.001
Employed	12,071 (57.2)	1,631 (13.5)	10,440 (86.5)	1.065; 0.943–1.202; *p* = 0.311	11,131 (92.2)	940 (7.8)	0.500; 0.441–0.568; *p* < 0.001
Unemployed	3,194 (15.1)	437 (13.7)	2,757 (86.3)	1	2,744 (85.9)	450 (14.1)	1
**Marital status**							
Single	9,558 (45.3)	1,490 (15.6)	8,068 (84.4)	0.840; 0.763–0.925; *p* < 0.001	8,571 (89.7)	987 (10.3)	1.278; 0.141–1.430; *p* < 0.001
Has had a relationship	11,548 (54.7)	1,477 (12.8)	10,071 (87.2)	1	10,638 (92.1)	910 (7.9)	1
**Country income**							
LIC	509 (2.4)	33 (6.5)	476 (93.5)	0.491; 0.340–0.708; *p* < 0.001	465 (91.4)	44 (8.6)	1.476; 1.057–2.061; *p* = 0.022
LMIC	11,388 (54.0)	1,979 (17.4)	9,409 (82.6)	1.477; 1.333–1.636; *p* < 0.001	10,188 (89.5)	1,200 (10.5)	1.722; 1.505–1.971; *p* < 0.001
UMIC	4,166 (19.7)	365 (8.8)	3,801 (91.2)	0.754; 0.655–0.867; *p* < 0.001	3,813 (91.5)	353 (8.5)	1.462; 1.243–1.720; *p* < 0.001
HIC	5,043 (23.9)	590 (11.7)	4,453 (88.3)	1	4,743 (94.1)	300 (5.9)	1
Stringency index	47.3 (57.8)	45.9 (27.7)	47.5 (27.8)	1.002; 1.001–1.004; *p* = 0.009	47.4 (27.5)	45.3 (29.9)	0.999; 0.997–1.000; *p* = 0.112
18–24	5,182 (24.6)	134 (2.6)	5,048 (97.4)	1.733; 1.156–2.600; *p* = 0.008	4,297 (82.9)	885 (17.1)	0.847; 0.721–0.995; *p* = 0.044
25–49	12,807 (60.7)	506 (4.0)	12,301 (96.0)	2.284; 1.651–3.161; *p* < 0.001	9,905 (77.3)	2,902 (22.7)	1.141; 1.016–1.282; *p* = 0.026
50+	3,117 (14.8)	48 (1.5)	3,069 (98.5)	1	2,598 (83.3)	519 (16.7)	1
**Sex**							
Females	13,052 (61.8)	317 (2.4)	12,735 (97.6)	0.523; 0.447–0.612; *p* < 0.001	10,726 (82.2)	2,326 (17.8)	0.677; 0.631–0.726; *p* < 0.001
Males	8,054 (38.2)	371 (4.6)	7,683 (95.4)	1	6,074 (75.4)	1,980 (24.6)	1
**Education level**							
No formal Educ	400 (1.9)	19 (4.8)	381 (95.3)	1.310; 0.806–2.129; *p* = 0.275	342 (85.5)	58 (14.5)	0.428; 0.321–0.571; *p* < 0.001
Primary	479 (2.3)	30 (6.3)	449 (93.7)	2.133; 1.443–3.154; *p* < 0.001	390 (81.4)	89 (18.6)	0.709; 0.559–0.901; *p* = 0.005
Secondary	3,635 (17.2)	157 (4.3)	3,478 (95.7)	1.753; 1.442–2.133; *p* < 0.001	2,824 (77.7)	811 (22.3)	1.258; 1.146–1.382; *p* < 0.001
University	16,592 (78.6)	482 (2.9)	16,110 (97.1)	1	13,244 (79.8)	3,348 (20.2)	1
**Employment status**							
Retiree	846 (4.0)	10 (1.2)	836 (98.8)	0.492; 0.246–0.985; *p* = 0.045	744 (87.9)	102 (21.1)	0.425; 0.333–0.543; *p* < 0.001
Student	4,995 (23.7)	101 (2.0)	4,894 (98.0)	0.382; 0.284–0.514; *p* < 0.001	4,219 (84.5)	776 (15.5)	0.476; 0.418–0.542; *p* < 0.001
Employed	12,071 (57.2)	427 (3.5)	11,644 (96.5)	0.772; 0.630–0.946; *p* = 0.012	9,528 (78.9)	2,543 (21.1)	0.663; 0.602–0.729; *p* < 0.001
Unemployed	3,194 (15.1)	150 (4.7)	3,044 (95.3)	1	2,309 (72.3)	885 (27.7)	1
**Marital status**							
Single	9,558 (45.3)	338 (3.5)	9,220 (96.5)	1.361; 1.144–1.619; *p* = 0.001	7,538 (78.9)	2,020 (21.1)	1.186; 1.094–1.286; *p* < 0.001
Has had a relationship	11,548 (54.7)	350 (3.0)	11,198 (97.0)	1	9,262 (80.2)	2,286 (19.8)	1
**Country income**							
LIC	509 (2.4)	10 (2.0)	499 (98.0)	0.426; 0.223–0.815; *p* = 0.010	399 (78.4)	110 (21.6)	1.848; 1.466–2.328; *p* < 0.001
LMIC	11,388 (54.0)	440 (3.9)	10,948 (96.1)	1.094; 0.907–1.320; *p* = 0.347	8,488 (74.5)	2,900 (25.5)	2.690; 2.435–2.970; *p* < 0.001
UMIC	4,166 (19.7)	67 (1.6)	4,099 (98.4)	0.426; 0.319–0.570; *p* < 0.001	3,452 (82.9)	714 (17.1)	1.602; 1.420–1.808; *p* < 0.001
HIC	5,043 (23.9)	171 (3.4)	4,872 (96.6)	1	4,461 (88.5)	582 (11.5)	1
Stringency index	47.3 (57.8)	52.2 (26.8)	47.1 (27.8)	1.012; 1.009–1.015; *p* < 0.001	47.3 (27.4)	46.9 (29.3)	1.003; 1.002–1.004; *p* < 0.001
18–24	5,182 (24.6)	864 (16.7)	4,318 (83.3)	1.014; 0.859–1.196; *p* = 0.873	2,393 (46.2)	2,789 (53.8)	1.046; 0.921–1.188; *p* = 0.488
25–49	12,807 (60.7)	2,313 (18.1)	10,494 (81.9)	1.149; 1.021–1.293; *p* = 0.022	5,156 (40.3)	7,651 (59.7)	0.998; 0.910–1.094; *p* = 0.964
50+	3,117 (14.8)	533 (17.1)	2,584 (82.9)	1	1,298 (41.6)	1,819 (58.4)	1
**Sex**							
Females	13,052 (61.8)	2,212 (16.9)	10,840 (83.1)	0.898; 0.834–0.967; *p* = 0.005	5,835 (44.7)	7,217 (55.3)	1.274; 1.202–1.351; *p* < 0.001
Males	8,054 (38.2)	1,498 (18.6)	6,556 (81.4)	1	3,012 (37.4)	5,042 (62.6)	1
**Education level**							
No formal Educ	400 (1.9)	73 (18.3)	327 (81.8)	1.181; 0.907–1.538; *p* = 0.218	94 (23.5)	306 (76.5)	0.486; 0.383–0.618; *p* < 0.001
Primary	479 (2.3)	117 (24.4)	362 (75.6)	1.683; 1.356–2.088; *p* < 0.001	176 (36.7)	303 (63.3)	0.928; 0.766–1.125; *p* = 0.446
Secondary	3,635 (17.2)	735 (20.2)	2,900 (79.8)	1.305; 1.186–1.437; *p* < 0.001	1,705 (46.9)	1,930 (53.1)	1.134; 1.051–1.225; *p* = 0.001
University	16,592 (78.6)	2,785 (16.8)	13,807 (83.2)	1	6,872 (41.4)	9,720 (58.6)	1
**Employment status**							
Retiree	846 (4.0)	155 (18.3)	691 (81.7)	1.162; 0.931–1.450; *p* = 0.185	366 (43.3)	480 (56.7)	0.853; 0.717–1.014; *p* = 0.071
Student	4,995 (23.7)	847 (17.0)	4,148 (83.0)	1.085; 0.944–1.248; *p* = 0.251	2,355 (47.1)	2,640 (52.9)	0.973; 0.875–1.082; *p* = 0.615
Employed	12,071 (57.2)	2,183 (18.1)	9,888 (81.9)	1.162; 1.041–1.297; *p* = 0.007	4,751 (39.4)	7,320 (60.6)	0.845; 0.777–0.919; *p* < 0.001
Unemployed	3,194 (15.1)	525 (16.4)	2,669 (83.6)	1	1,375 (43.3)	1,819 (57.0)	1
**Marital status**							
Single	9,558 (45.3)	1,661 (17.4)	7,897 (82.6)	1.006; 0.923–1.096; *p* = 0.897	4,299 (45.0)	5,259 (55.0)	1.210; 1.132–1.295; *p* < 0.001
Has had a relationship	11,548 (54.7)	2,049 (17.7)	9,499 (82.3)	1	4,548 (39.4)	7,000 (60.6)	1
**Country income**							
LIC	509 (2.4)	81 (15.9)	428 (84.1)	0.796; 0.620–1.023; *p* = 0.075	203 (39.9)	306 (60.1)	0.709; 0.587–0.856; *p* < 0.001
LMIC	11,388 (54.0)	1,968 (17.3)	9,420 (82.7)	0.993; 0.908–1.087: *p* = 0.882	4,099 (36.0)	7,289 (64.0)	0.592; 0.551–0.635; *p* < 0.001
UMIC	4,166 (19.7)	754 (18.1)	3,412 (81.9)	0.966; 0.866–1.078; *p* = 0.538	2,100 (50.4)	2,066 (49.6)	1.011; 0.929–1.100; *p* = 0.800
HIC	5,043 (23.9)	907 (18.0)	4,136 (82.0)	1	2,445 (48.5)	2,598 (51.5)	1
Stringency index	47.3 (57.8)	50.5 (27.1)	46.6 (27.9)	1.005; 1.004–1.007; *p* < 0.001	49.3 (28.3)	45.8 (27.3)	1.002; 1.001–1.003; *p* = 0.001
18–24	5,182 (24.6)	514 (9.9)	4,668 (90.1)	0.532; 0.446–0.635; *p* < 0.001	1,951 (37.6)	3,231 (62.4)	3.224; 2.762–3.763; *p* < 0.001
25–49	12,807 (60.7)	2,056 (16.1)	10,751 (83.9)	0.814; 0.728–0.910; *p* < 0.001	3,540 (27.6)	9,267 (72.4)	2.241; 1.982–2.533; *p* < 0.001
50+	3,117 (14.8)	705 (22.6)	2,412 (77.4)	1	472 (15.1)	2,645 (84.9)	1
**Sex**							
Females	13,052 (61.8)	2,249 (17.2)	10,803 (82.8)	1.507; 1.389–1.637; *p* < 0.001	4,081 (31.3)	8,971 (68.7)	1.320; 1.235–1.411; *p* < 0.001
Males	8,054 (38.2)	1,026 (12.7)	7,028 (87.3)	1	1,882 (23.4)	6,172 (76.6)	1
**Education level**							
No formal Educ	400 (1.9)	99 (24.8)	301 (75.3)	2.198; 1.723–2.804; *p* < 0.001	301 (75.3)	99 (24.8)	6.445; 5.050–8.226; *p* < 0.001
Primary	479 (2.3)	106 (22.1)	373 (77.9)	1.723; 1.372–2.163; *p* < 0.001	261 (54.5)	218 (45.5)	3.313; 2.731–4.020; *p* < 0.001
Secondary	3,635 (17.2)	513 (14.1)	3,122 (85.9)	1.033; 0.926–1.152; *p* = 0.563	1,251 (34.4)	2,384 (65.6)	1.261; 1.161–1.371; *p* < 0.001
University	16,592 (78.6)	2,557 (15.4)	14,035 (84.6)	1	4,150 (25.0)	12,442 (75.0)	1
**Employment status**							
Retiree	846 (4.0)	218 (25.8)	628 (74.2)	1.562; 1.267–1.925; *p* < 0.001	139 (16.4)	707 (83.6)	0.496; 0.397–0.620; *p* < 0.001
Student	4,995 (23.7)	572 (11.5)	4,423 (88.5)	1.404; 1.197–1.646; *p* < 0.001	1,660 (33.2)	3,335 (66.8)	0.526; 0.470–0.588; *p* < 0.001
Employed	12,071 (57.2)	2,051 (17.0)	10,020 (83.0)	1.374; 1.219–1.547; *p* < 0.001	2,704 (22.4)	9,367 (77.6)	0.460; 0.422–0.502; *p* < 0.001
Unemployed	3,194 (15.1)	434 (13.6)	2,760 (86.4)	1	1,460 (45.7)	1,734 (54.3)	1
**Marital status**							
Single	9,558 (45.3)	1,086 (11.4)	8,472 (88.6)	0.681; 0.620–0.749; *p* < 0.001	3,001 (31.4)	6,557 (68.6)	0.949; 0.880–1.024; *p* < 0.177
Has had a relationship	11,548 (54.7)	2,189 (19.0)	9,359 (81.0)	1	2,962 (25.6)	8,586 (74.4)	1
**Country income**							
LIC	509 (2.4)	108 (21.2)	401 (78.8)	1.239; 0.985–1.559; *p* = 0.067	108 (21.2)	401 (78.8)	0.682; 0.542–0.856; *p* = 0.001
LMIC	11,388 (54.0)	1,540 (13.5)	9,848 (86.5)	0.899; 0.817–0.989; *p* = 0.029	3,496 (30.7)	7,892 (69.3)	1.055; 0.974–1.142; *p* = 0.189
UMIC	4,166 (19.7)	764 (18.3)	3,402 (81.7)	1.035; 0.925–1.157; *p* = 0.550	1,040 (25.0)	3,126 (75.0)	0.844; 0.764–0.931; *p* = 0.001
HIC	5,043 (23.9)	863 (17.1)	4,180 (82.9)	1	1,319 (26.2)	3,724 (73.8)	1
Stringency index	47.3 (57.8)	52.1 (26.7)	46.3 (27.9)	1.006; 1.005–1.008; *p* < 0.001	49.8 (27.1)	46.3 (28.0)	1.007; 0.006–1.008; *p* < 0.001

Table 1 shows that respondents 25–49 years old had significantly higher odds of increase in sexual activity (AOR: 2.651; 95% CI: 2.217–3.169; *p* < 0.001), housing insecurity (AOR: 2.284; 95% CI:1.651–3.161; *p* < 0.001), food insecurity (AOR: 1.141; 95% CI: 1.016–1.282; *p* = 0.026), worsening of family relationships (AOR: 1.149; 95% CI: 1.021–1.293; *p* = 0.022), and post-traumatic stress syndrome (AOR: 2.241; 95% CI: 1.982–2.533; *p* < 0.001) than respondents ≥50 years during the COVID-19 pandemic. In addition, they had significantly lower odds of feeling socially isolated (AOR: 0.842; 95% CI: 0.767–0.925; *p* < 0.001), change in sleep pattern (AOR: 0.822; 95% CI: 0.741–0.913; *p* < 0.001), and memory complaints (AOR: 0.814; 95% CI: 0.728–0.910; *p* < 0.001) than respondents ≥50 years during the COVID-19 pandemic. There were no significant differences in emotional distress and access to financial support among all age groups.

## Discussion

Our study highlights mental health and wellbeing in different age groups during the COVID-19 pandemic, with different patterns observed in each age group. Younger individuals seemed more likely to have reported an increase in sexual activity, housing insecurity, and post-traumatic stress symptoms during the pandemic. In contrast, they had lower odds of feeling socially isolated and had fewer memory complaints. Interestingly, there were no age differences in the odds of emotional distress or financial insecurity during the pandemic. The findings supported the study hypothesis that posited that mental health and wellbeing would vary across these age groups.

Specifically, this study adds to the literature by offering a nuanced, age-stratified analysis of wellbeing and mental health outcomes using data from over 21,000 participants across 152 countries—one of the largest such global datasets available for this period. It provides evidence of age-related differences in both risk and protective factors for a greater representation of global experiences during the COVID-19 pandemic. In addition, the study utilized a comprehensive survey tool available in multiple languages, ensuring inclusivity. The incorporation of various social media platforms and networks for data collection contributed to a broad and varied participant pool. The survey tool was validated, thereby ensuring the validity of data.

The study, however, had some limitations. For example, the use of an online survey for data collection introduces a risk of selection bias, as only participants with internet access and proficiency in survey languages were included. This limits the generalizability of findings to populations with different attributes. In addition, the study relied primarily on self-reported data, which might have introduced some recall bias and social desirability bias, potentially impacting responses about changes in sexual activity or emotional distress due to stigma or socially undesirable experiences. In addition, the cross-sectional nature of the study restricts the establishment of causal relationships between variables, which needs longitudinal studies. Furthermore, there was a moderate positive skew in the age distribution. The categorization of the age variable helped to mitigate the influence of the right-skewed distribution by reducing the impact of extreme values in the higher age range, thereby improving the robustness of statistical analyses. Also, by using age as a categorical rather than continuous variable in the logistic regression models, the analysis avoided assumptions of normality and linearity. This approach allowed for a clearer interpretation of the associations between specific age groups and the outcome variable, facilitating the identification of age-specific trends and risk patterns. The logistic regression, being a non-parametric method that does not assume normal distribution of independent variables, is well-suited to handle such skewed data, further enhancing the reliability of the findings. Despite these limitations, the study offers valuable insights into the mental health and wellbeing of diverse populations during the COVID-19 pandemic, thus contributing to the global understanding of this critical public health issue.

First, the findings align with social theories that underscore the role of age-related experiences and societal expectations. This alignment provides a nuanced understanding of the disparities in mental health across different age groups during the COVID-19 pandemic. Social theories often posit that individuals undergo unique challenges and expectations in distinct life stages, and these factors can significantly influence mental wellbeing (38–40). Our findings are like prior studies indicating that younger individuals had more significant mental health challenges during the pandemic (37, 41, 87–90). This might be attributed to disruptions in school, education, employment, and social connections (25, 55, 91) as well as worsening relationships with self and limited capacity for self-care (92). Young people facing restricted access to outdoor social activities may be more prone to anxiety or depression, and those possessing psychological traits such as emotional reactivity and experiential avoidance might face an increased risk of experiencing poor mental health (55, 93, 94). Prolonged engagement with social media and screens during the pandemic also increased the risk of body dysmorphia disorder with its attendant psychological impact (95). The restriction, however, had some positive impacts on young people, as some developed a greater capacity for self-care and reflection upon their lives (92).

The findings also seem to indicate age variability in mental health challenges during the pandemic. Younger individuals seem to be more prone to experiencing PTSS than older individuals, though there were no significant age differences with the experience of emotional distress. The higher risk of PTSS aligns with previous research predating the pandemic, demonstrating that younger individuals tend to experience PTSS more frequently than older individuals (41, 96, 97). Country-level evidence also supports the notion that PTSS is more prevalent among younger individuals during the pandemic (36), a trend observed in past disasters (97, 98). The higher risk of COVID-19-related PTSS among younger individuals may be due to age-related vulnerability to trauma exposure. Middle-aged adults face the highest risk of trauma exposure (99, 100) and may be less equipped to cope with traumatic events, especially those who are from poor households (101). In addition, older individuals may be more resilient and adaptive to similar events (102). Moreover, emotional regulation in older individuals allows them to withstand stress better (103).

This risk of emotional distress might have been ameliorated by increased sexual activity, as mental health improved with sexual activity during the pandemic (104). While the pandemic might have intensified the risk of mental health challenges among the younger population, increased sexual activity might have been a mitigating factor, reducing the risk of emotional distress. An increase in sexual activity results in better dyadic cohesion, communication, and satisfaction that consequently reduce distress and depression (104). However, this proposed protective effect seemed to be specific to emotional distress and not necessarily extend to PTSS. To further advance our understanding of these complex interactions, future studies are recommended to explore this intricate interplay between pandemic-related factors, sexual activity, and distinct mental health outcomes.

Second, our findings suggest that younger individuals appeared to fare better than older adults during the pandemic, particularly about lower levels of social isolation, food insecurity, and memory complaints. This outcome may be partially explained by their greater engagement with digital technologies. Younger populations are generally more digitally connected, a factor that facilitated virtual interactions, sustained social ties, and access to peer support during lockdowns and social distancing mandates. Previous studies have highlighted the protective effect of online platforms in maintaining psychosocial wellbeing, showing that virtual socialization and online networking can significantly mitigate the adverse effects of isolation during crises (105, 106). Indeed, digital literacy and frequent engagement with online communities have been linked to greater psychological resilience during the COVID-19 pandemic (28, 107, 108).

In addition to digital connectivity, younger individuals may benefit from developmental and cognitive factors that enhance their adaptability. Research shows that younger adults tend to adopt more optimistic appraisals of adversity, which is associated with better psychological outcomes during periods of uncertainty (109). Optimism, in turn, is known to foster proactive coping strategies, such as seeking social support, reframing challenges, and sustaining hope—all of which are beneficial in times of collective trauma. Moreover, younger individuals are often more flexible and responsive to change, which may account for their capacity to manage stressors more effectively during public health emergencies. This adaptability was supported by studies indicating that younger people are more likely to use psychological coping mechanisms and maintain social networks as buffers against stress during disasters (110).

Another contributing factor may lie in age-related differences in emotional processing. The “positivity effect” in older adults—a well-documented phenomenon wherein older individuals preferentially attend to and remember positive over negative information—may paradoxically limit their ability to fully process and respond to emerging threats during a crisis (111). Conversely, younger adults are more likely to engage with a broader range of emotional content, including negative information, which could enhance situational awareness and adaptive response during a pandemic. This heightened engagement with information, when balanced with psychological resilience and social support, may have afforded younger individuals a cognitive advantage in navigating the complexities of the COVID-19 experience.

Third, the worsening of family relationships among individuals aged 25–49 years observed in this study is particularly concerning, as this age group represents the core of the economically productive population and is often responsible for the financial and emotional wellbeing of their households. Prior research has consistently shown that strong family support serves as a protective factor against mental health challenges, while inadequate family support is associated with increased psychological distress, anxiety, and depression (36, 112). In our study, the deterioration in familial relationships within this age group may be closely tied to increased stressors arising from housing and food insecurity, both of which were exacerbated by the economic consequences of the COVID-19 pandemic.

This age group constitutes the economically productive segment that may have been severely affected by the economic crisis linked to the pandemic (113). Economic instability, including job loss, reduced income, and heightened financial strain, disproportionately impacted adults in their working years during the pandemic, particularly in low- and middle-income settings (114, 115), and in Africa (116). Similarly, the World Bank reported that the global economic downturn led to rising levels of household poverty, disrupting the social fabric and straining interpersonal relationships within families (117). These stressors likely contributed to increased tension, conflict, and emotional withdrawal, all of which can undermine family cohesion and perceived support (24).

Our earlier findings align with these patterns, indicating that individuals who experienced wage losses during the pandemic reported no improvement—and in many cases deterioration—in family relationships (22). Moreover, stress-induced family dysfunction can have long-term implications for emotional regulation (118), particularly in households with children and adolescents, compounding the intergenerational impact of the pandemic's economic toll (119). Together, these findings underscore the importance of addressing economic vulnerabilities in tandem with psychosocial interventions. Strategies aimed at improving family resilience must, therefore, consider both the structural determinants of health—such as employment and housing stability—and relational dynamics within the household that are critical to emotional wellbeing.

Fourth, there were no significant differences in perceived financial security by age. Although the uniformity across age groups may appear counterintuitive—given that age often correlates with employment stability and financial responsibilities—this finding aligns with studies suggesting that the pandemic created widespread economic uncertainty that cut across demographic lines. From younger adults facing disrupted job entry and education (120) to older adults experiencing retirement insecurity or job loss (121), the sense of financial vulnerability became a near-universal concern. However, the impact of perceived financial insecurity may vary by age, particularly in terms of psychological and relational consequences. Middle-aged individuals, often described as the “sandwich generation,” (122) juggle multiple responsibilities—caring for children, supporting aging parents, and sustaining employment—all of which are susceptible to disruption under financial strain. Middle-aged adults reported elevated levels of psychological distress during the pandemic due to this convergence of stressors (123).

We hypothesized that inadequate financial security contributes to the deterioration of family relationships as financial insecurity has been directly linked to tension and conflict in family dynamics (124). Perceived financial strain, even if uniformly felt, may have differential effects on middle-aged adults due to their unique life-stage pressures. This implies that the wellbeing of the middle-aged cohort during the COVID-19 pandemic may be influenced by a distinct set of stressors. These stressors could be linked to the intricate balance between work, family responsibilities, and financial concerns. Food and housing insecurity—closely tied to financial wellbeing—have been strongly associated with poor mental health outcomes and negative family functioning (125). These insecurities, when compounded by reduced access to social services during lockdowns, likely intensified feelings of helplessness and interpersonal strain among this cohort. This underscores the need for age-sensitive, targeted interventions that address the structural vulnerabilities of middle-aged adults. Policies aimed at enhancing economic stability—such as wage support, childcare subsidies, and housing security—are not only critical for individual wellbeing but also for sustaining family resilience during prolonged crises. Interventions should also integrate mental health support tailored to those.

Fifth, we observed a potential age-related variation in the impact of the COVID-19 pandemic on sleep disturbances. Sleep health was significantly disrupted across age groups during the pandemic, but the underlying causes and consequences varied. Several studies have suggested that the physiological effects of SARS-CoV-2 may involve the central nervous system, potentially altering sleep patterns directly, particularly in older adults who may be more neurologically vulnerable due to age-related changes in brain function and immune regulation (126, 127). Moreover, older individuals often experience pre-existing sleep disorders such as insomnia or fragmented sleep, which may predispose them to immune dysregulation (128), thereby increasing susceptibility to COVID-19 infection and severe outcomes (129). This bidirectional relationship between poor sleep and vulnerability to infection warrants deeper investigation, particularly in aging populations.

Conversely, middle-aged adults may demonstrate greater resilience to sleep disturbances due to more established coping mechanisms, adaptive stress management strategies, and consistent daily routines (130). These protective factors might buffer the psychosocial impacts of the pandemic and help maintain more stable sleep patterns in this group. However, even among middle-aged individuals, sustained exposure to pandemic-related stressors—including job insecurity, caregiving burdens, and health concerns—could still disrupt sleep, albeit in less overt or chronic ways. The possibility that sleep disruption during pandemics exacerbates systemic inflammation, which in turn may contribute to mental health deterioration, creates a concerning feedback loop that must be addressed (131).

Emerging evidence also highlights that individuals with pre-existing conditions such as obstructive sleep apnea may experience worsened sleep quality during pandemics and face increased risk of severe COVID-19 complications (132). This intersection between physical and mental vulnerability underscores the need for integrated care approaches that consider sleep health as part of pandemic response planning.

Further research is necessary to examine the drivers of sleep pattern changes across different age groups, particularly in middle-aged individuals whose experience may be nuanced and under-reported in current literature. Longitudinal and mixed-methods studies could provide insight into how coping strategies evolve and the role of mediating factors such as employment status, family dynamics, and digital technology use. Moreover, data disaggregation by gender, socioeconomic status, and cultural context could enrich the understanding of sleep-related disparities during public health emergencies.

This study emphasizes that mental health outcomes during the pandemic are shaped by a complex interplay of age, social dynamics, and external stressors. For example, while younger adults reported higher levels of anxiety and post-traumatic stress symptoms due to social isolation and educational disruptions, older adults faced grief, health anxieties, and concerns about medical access. Understanding the age-specific psychosocial burdens is critical to designing comprehensive and equitable mental health interventions. Policymakers and mental health support systems must adopt a life-course perspective in addressing these issues. Tailored interventions—such as housing security programs for youth, trauma-informed care for middle-aged caregivers, and social connection initiatives for the older adult—can help mitigate sleep disturbances and mental health burdens exacerbated by the pandemic. In addition, integrating sleep assessments into routine mental health evaluations and creating accessible digital tools for sleep hygiene education may offer scalable solutions during times of crisis.

## Conclusion

This study highlights age as a key factor influencing mental health during the first wave of COVID-19 with distinct age-specific patterns: younger adults faced more sexual activity changes, housing insecurity, and post-traumatic stress symptoms but less social isolation and food insecurity; middle-aged adults experienced more family strain and food insecurity; older adults showed greater overall wellbeing. Despite shared emotional and financial distress across all ages, the findings underscore the need for age-tailored mental health responses. Interventions should address specific stressors like housing, food insecurity, and relationship strain. Longitudinal research is needed to inform future age-responsive mental health policies.

## Data Availability

The raw data supporting the conclusions of this article will be made available by the authors, without undue reservation.
